# Iodine-125 brachytherapy for orbital-invasive low-grade myxofibrosarcoma of the maxillary sinus: a case report challenging conventional therapeutic paradigms

**DOI:** 10.3389/fonc.2025.1590061

**Published:** 2025-10-15

**Authors:** Qiyu Sun, Shuai Li, Quanli Qiu, Yanbo Hu, Rui Huang, Yan Chen, Zhenzhen Cui, Jiaxin Yang, Xiaowen Ma, Min Li

**Affiliations:** ^1^ The Postgraduate Training Base of Jinzhou Medical University (The 960th Hospital of PLA), Jinan, China; ^2^ The 960th Hospital of People's Liberation Army (PLA), Jinan, Shandong, China

**Keywords:** low-grade myxofibrosarcoma, iodine-125 brachytherapy, orbital-invasive, case report, CT-guided

## Abstract

Low-grade myxofibrosarcoma (LGMFS) is a rare subtype of soft tissue sarcoma, with an estimated incidence of approximately 0.18 cases per million population, accounting for about 0.6% of all soft tissue sarcomas. It is characterized by a high local recurrence rate and malignant potential. Orbital involvement secondary to maxillary sinus LGMFS represents an exceptionally rare occurrence. The management of such cases is complicated by anatomical complexity (e.g., proximity to optic nerve, lacrimal apparatus, and orbital vasculature) and limitations of conventional therapies, including radical resection combined with chemoradiation. In this anatomically complex case of recurrent Low-Grade Myxofibrosarcoma, iodine-125 brachytherapy achieved durable local control (12-month progression-free survival), demonstrating superior precision targeting and reduced systemic toxicity compared to conventional therapies. No acute radiation toxicity was observed, while facial swelling and eye pain were relieved with concurrent improvement in visual function.

## 1 Introduction

Low-Grade Fibromyxoid Sarcoma (LGFMS) is a rare soft tissue sarcoma, with an incidence of approximately 0.18 per million population, accounting for 0.6% of all soft tissue sarcomas. It predominantly affects young adults, primarily involving the deep soft tissues of the extremities (especially the thighs and shoulders) and trunk (1). Rare cases may occur in unusual sites such as the head and neck, paranasal sinuses, or breast (2). Characterized by aggressive local invasiveness and high recurrence rates (40%–60%), LGFMS exhibits a distant metastasis risk of less than 10% at initial diagnosis. Despite its histological classification as “low-grade,” the tumor demonstrates infiltrative growth patterns and occult satellite lesions, leading to frequent recurrences (3). These features significantly increase the risk of patient disability and transformation into high-grade sarcoma, posing a critical challenge in clinical management. Standard treatment primarily involves surgical resection, with adjuvant radiotherapy or chemotherapy recommended postoperatively to reduce recurrence risk (4, 5). The management of maxillary sinus LGFMS presents particular challenges due to its unique anatomical constraints and exceptional rarity (6, 7). Although surgical intervention remains the primary approach for recurrent cases (5), managing recurrences in the maxillary sinus with orbital involvement is particularly challenging due to: 1) Surgical limitations due to adjacent critical structures (surgical manipulation may risk damage to the optic nerve and retina); 2)Risk of cumulative radiation toxicity from radiotherapy equipment (radiation-induced optic neuropathy; radiation-induced cerebral edema); and 3) reduced tolerance for systemic therapies.Iodine-125 (I-125I) brachytherapy offers precise, minimally invasive continuous low-dose-rate radiation delivery (8), particularly suitable for inoperable, post-radiation recurrent, or metastatic solid tumors (9). To address these clinical dilemmas, we propose I-125 brachytherapy as a novel therapeutic option, precise radiotherapy control for recurrent cases unsuitable for surgical intervention or conventional chemoradiotherapy.

## 2 Case report

### 2.1 Relevant past interventions

A 28-year-old female presented with nasal swelling in September 2022. MRI revealed a space-occupying lesion in the left maxillary sinus with maxillary bone invasion. She underwent initial surgical resection in October 2022. Tumor margins and lymph nodes (zone 2, 0/4) were negative, with a total tumor size of 4.5×4.5×3 cm. She received adjuvant radiotherapy (33 fractions) and showed good recovery during follow-up. In August 2023 (11 months post-surgery), she developed recurrent swelling at the primary site unresponsive to anti-inflammatory therapy. Mucosal biopsy confirmed tumor recurrence, and a second R0 resection of the left sinonasal tumor was performed in September 2023. Adjuvant chemotherapy was initiated 4 weeks postoperatively: Doxorubicin 60 mg/m²Cisplatin 75 mg/m² (Day 1); Ifosfamide 1.2 g/m² (Days 1–5); Repeated every 21 days. The patient completed 5 cycles of treatment but discontinued it due to severe chemotherapy - related toxicities, including nausea, vomiting, neutropenia, thrombocytopenia, and alopecia.

### 2.2 MRI and CT examination

**Figure 1:**
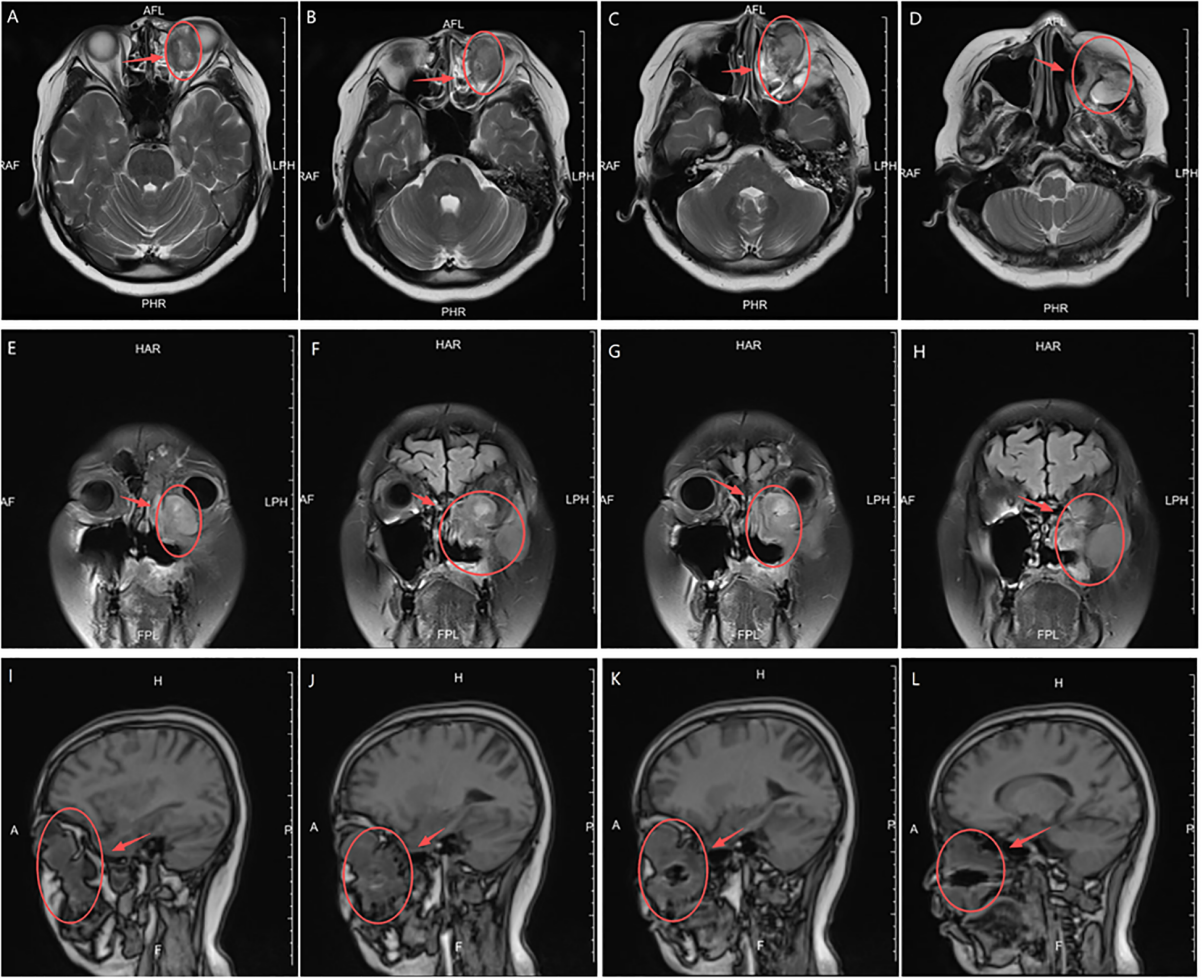
MRI Anatomical imaging: Findings consistent with (LGMFS) tumor recurrence involving the orbit, (as indicated by the arrows). **(A–D)** Axial T2-weighted image. **(E–H)** Coronal Short Tau Inversion Recovery (STIR) sequence image. **(I–L)** Sagittal T1-weighted image.

**Figure 2:**
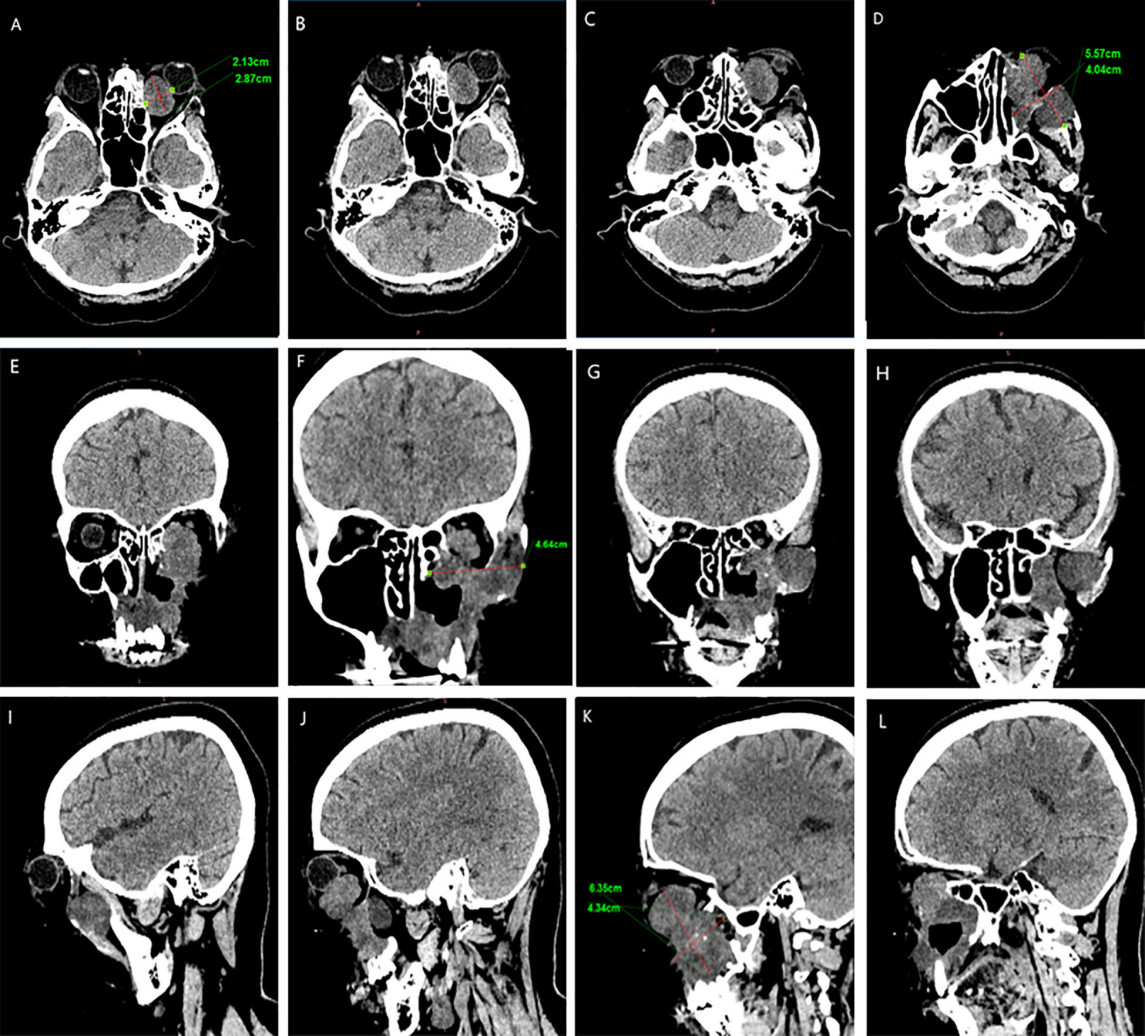
CT Anatomical imaging: Findings consistent with (LGMFS) tumor recurrence involving the orbit. **(A–D)** Axial CT image. **(E–H)** Coronal CT image. **(I–L)** Sagittal CT image.

In February 2024, the patient presented with recurrent symptoms including left facial swelling, left orbital displacement, diplopia, progressive visual acuity deterioration, and ocular distension. MRI revealed local tumor recurrence, demonstrating superior growth through the superior wall of the maxillary sinus into the orbital cavity, with optic nerve compression and orbital bone destruction (**Figure 1**). CT findings suggest tumor recurrence. (**Figure 2**).

### 2.3 Treatment

**Figure 3:**
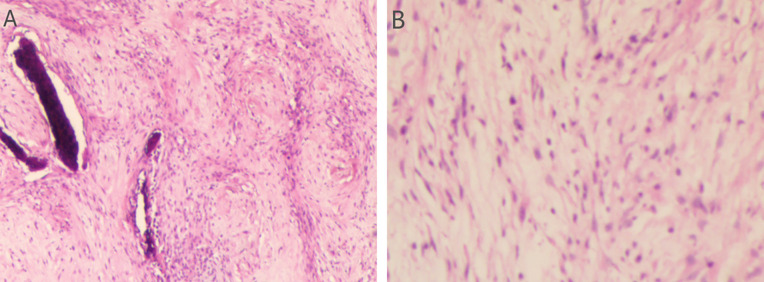
Assessment of the H&E stained tumor tissue. **(A)** The histopathological evaluation in lower magnification, seen in the left panel, the tissue exhibits a fascicular growth pattern. The cells possess elongated nuclei and include a significant number of spindle-shaped cells. Evident myxoid change is noted within the stroma. **(B)** In higher magnification, in the right panel, the section demonstrates a relatively uniform population of spindle-shaped cells. The nuclei are slender with evenly distributed chromatin. Mitotic figures are inconspicuous, suggesting low proliferative activity.

**Figure 4:**
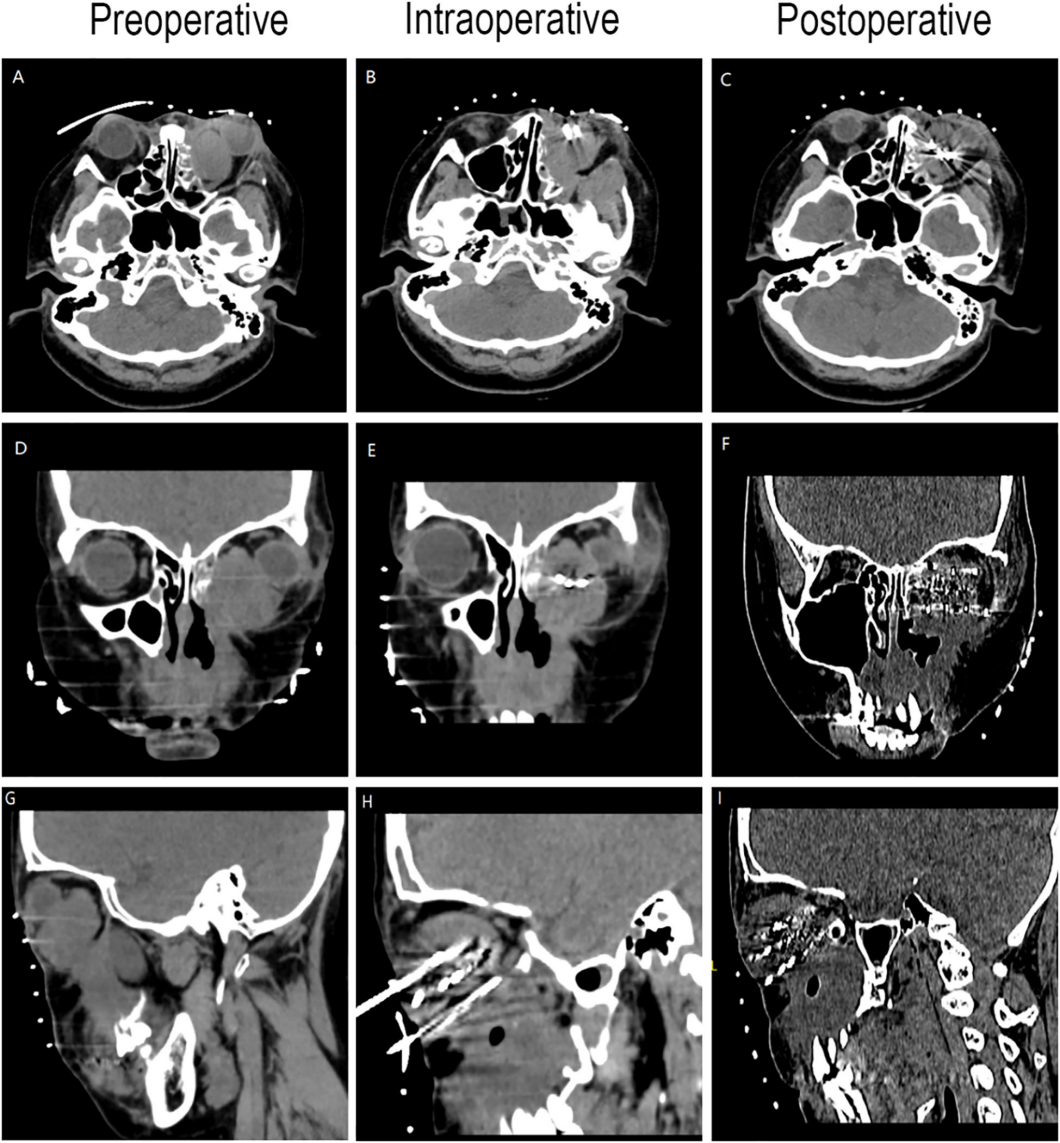
CT-guided iodine-125 seed implantation. **(A–C)** Axial CT image. **(D–F)** Coronal CT image. **(G–I)** Sagittal CT image.

Preoperative Pathological Diagnosis confirmed Low-Grade Myxofibrosarcoma (left maxillary sinus) with spindle cell morphology, fibrous histiocytoma-like architecture (**Figure 3**), and myxoid degeneration. Immunohistochemistry showed Vimentin (+), SATB2 (+), MDM-2 (+), and Ki-67 (+30%). Considering the patient’s treatment history and the limitations of the anatomical structure, we proposed the use of Iodine-125 particle implantation therapy. Given the unique location of the tumor (which directly invaded the orbital region and compressed the optic nerve), this form of brachytherapy inherently carries the risk of optic nerve and retinal damage, presenting a significant clinical challenge. Prior to the surgery, we extensively reviewed a large number of relevant studies and planned the surgical procedure using the Treatment Planning System (TPS)to ensure the scientific basis of the surgical plan and to minimize the safety risks to adjacent organs (such as the optic nerve) (10, 11). Based on the patient’s tolerable surgical duration, the treatment was divided into two stages. Preoperative TPS planning guided precise implantation of radioactive seeds before each procedure (**Supplementary Figures 1**, **2**). A total of 197 I-125 seeds (0.3 mCi/seed) were implanted to deliver a total prescription dose of 12,000 cGy (Stage 1: 71 seeds, Stage 2: 126 seeds). The initial surgery was performed in March 2024 (**Figure 4**). Postoperative CT-based TPS dosimetric evaluation confirmed high concordance between the spatial distribution/dosimetry of implanted I-125 seeds and preplanned configurations (**Supplementary Figures 3**, **4**). The preceding description summarizes the patient’s core clinical profile (**Table 1**). All treatment plans were generated using the domestic Fei Tian Program TPS, with its specialized Fei Tian Brachy v3.00.00 module for brachytherapy planning.

**Table 1:** Case timeline and key data.

Time	Event	Location	Symptoms/Signs	Treatment/Intervention
September 2022	Hospital visit due to nasal swelling	Maxillary sinus	Left maxillary sinus space-occupying lesion with invasion of the maxilla	Magnetic resonance imaging (MRI) examination
October 2022	Tumor resection	Maxillary sinus	Postoperative pathology: Low-Grade Myxofibrosarcoma	Extensive resection under general anesthesia
October-December 2022	Postoperative radiotherapy	Maxillary sinus	Recovery was good during the follow-up period	Adjuvant radiotherapy (33 sessions)
August 2023	Tumor recurrence	Maxillary sinus	Pathology: Low-Grade Myxofibrosarcoma	Nasal mucosa biopsy
September 2023	Tumor resection	Maxillary sinus	Major resection of the left maxilla	Extensive resection under general anesthesia
October 2023	Postoperative chemotherapy	Maxillary sinus	Treatment stopped due to severe chemotherapy-related toxic reactions	Completed 5 cycles of chemotherapy
February 2024	Tumor recurrence	Maxillary sinus with left orbital involvement	Left-sided facial swelling, diplopia, decreased vision, proptosis	MRI examination
March 2024	Iodine-125 seed implantation treatment	Maxillary sinus with left orbital involvement	Completed implantation of Iodine-125 seeds in two sessions	Iodine-125 seed implantation under local anesthesia

### 2.4 Follow up

**Figure 5:**
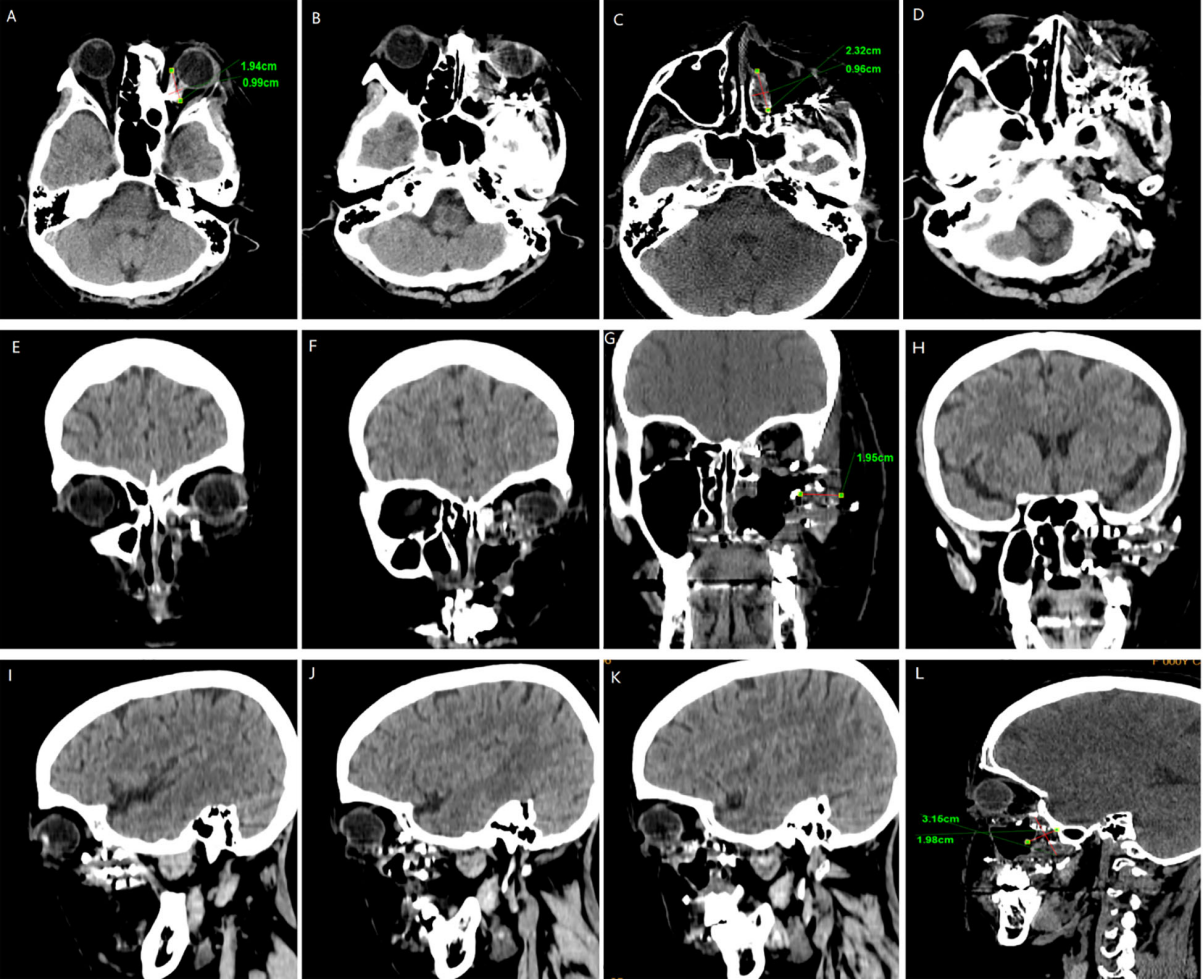
CT surveillance findings: **(A–D)** Axial CT image. **(E–H)** Coronal CT image. **(I–L)** Sagittal CT image (12-month follow-up).

**Figure 6:**
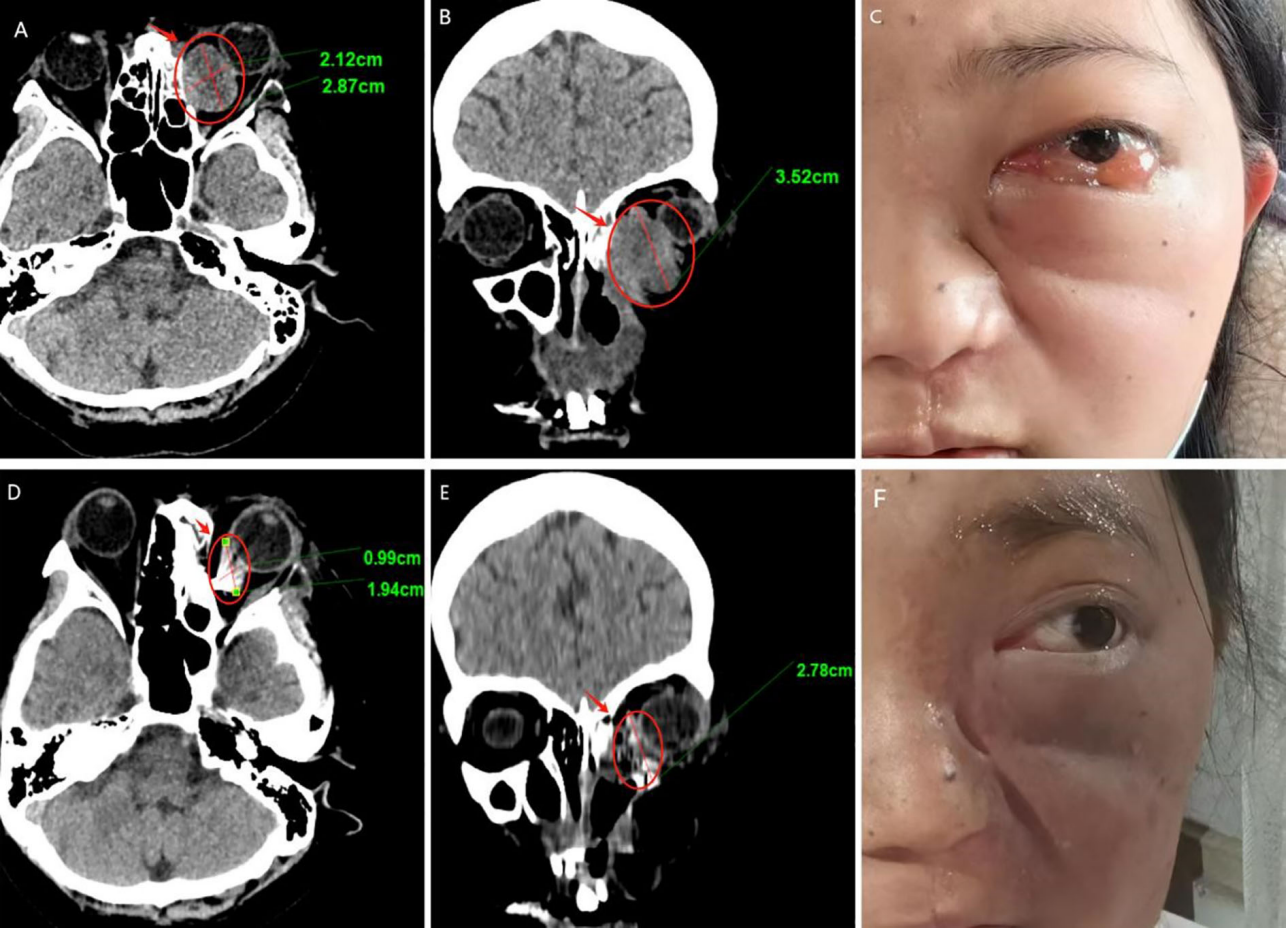
Follow-up results **(A–C)** Pre-therapeutic imaging series, **(D–F)** Post-therapeutic imaging series (12-month follow-up) **(A, D)** CT axial views, **(B, E)** CT coronal views.

We followed up with the patient at 1 month, 3 months, 6 months, and 1 year postoperatively. After one year of follow-up, the volume of the primary tumor significantly decreased (the tumor size was approximately 5.57×4.04×6.35 cm before treatment, and 2.32×0.96×3.15 cm after treatment) (**Figure 5**). The intraorbital tumor decreased in size from 2.87×2.12×3.52 cm to 1.94×0.99×2.78 cm.The compression of the optic nerve by the tumor was alleviated, and both visual field and vision showed signs of recovery. The proptosis of the eyeball was significantly improved. The swelling and deformity of the eye were markedly improved, resulting in a more natural facial appearance (**Figure 6**). The patient’s visual acuity improved significantly from 20/200 (LogMAR 1.0) preoperatively to 20/30 (LogMAR 0.2) at 1 year postoperatively. The patient’s quality of life was significantly enhanced. No complications such as radiation-induced cataracts, optic neuropathy, or cerebral edema were observed during the last follow-up in March 2025.

## 3 Discussion

Low-Grade Myxofibrosarcoma (LGMFS) is a rare, aggressive soft tissue sarcoma with a high local recurrence rat (7). LGMFS occurring in the maxillary sinus and invading the orbit is even more uncommon. Current evidence demonstrates that adjuvant therapy (e.g., chemoradiation) following negative surgical margins (R0 resection) significantly reduces local recurrence risk (12). However, in this case, the tumor was located in the maxillary region, adjacent to critical structures such as the orbit, skull base, and cavernous sinus, making it difficult to achieve negative surgical margins. Research by Professor Li Wengang’s team notes (13): “Fibrosarcoma presents challenges in determining precise surgical resection margins and carries a risk of hematogenous metastasis, leading to a high postoperative recurrence rate.” Despite undergoing two prior surgical resections, conventional external beam radiotherapy, and chemotherapy, this patient experienced recurrence with orbital invasion due to the tumor’s intrinsic or acquired resistance to traditional radiotherapy and chemotherapy, demonstrating the limitations of surgery and conventional chemoradiotherapy in treating advanced low-grade myxofibrosarcoma of the maxilla. Conventional radiotherapy also faces significant challenges in such cases. A Mayo Clinic research team studying radiotherapy for soft tissue sarcomas states (14): “Radiation therapy in anatomically constrained regions often fails to deliver tumoricidal doses, resulting in local control failure.” In this patient, tumor invasion of the orbit with optic nerve compression made conventional external beam radiotherapy extremely difficult, as the radiation tolerance of the optic nerve (a radiosensitive structure) is far below the dose required for effective fibrosarcoma control (60–70 Gy). Furthermore, the radiosensitivity of the skull base and brain tissue further limited the delivery of therapeutic radiation doses (15).

Iodine-125 (I-125) seed implantation therapy, as a precise radiotherapy technique, demonstrated significant advantages in treating this case of recurrent maxillary fibrosarcoma. Its core mechanism lies in the continuous release of low-energy gamma rays (27.4-35.5 keV), enabling sustained irradiation of tumor tissue while maximizing protection of adjacent critical structures. This makes it particularly suitable for tumors abutting radiosensitive organs. Compared to conventional external beam radiotherapy, I-125 seed implantation achieves spatiotemporal redistribution of the radiation dose distribution. In the spatial dimension, it achieves high conformity of the high-dose region to the tumor target volume. In the temporal dimension, continuous low-dose-rate irradiation induces irreversible double-strand DNA breaks in tumor cells. These spatiotemporal characteristics significantly enhance the therapeutic ratio, delivering a higher biologically effective dose to the tumor while sparing critical structures such as the optic nerve and brain tissue.

Prior research indicates that I-125 brachytherapy demonstrates efficacy and safety in treating skull base and orbital tumors (16, 17). This safety profile is particularly crucial for optic nerve preservation, as seed implantation enables rapid dose fall-off, maintaining the optic nerve dose within safe tolerance limits. In this specific case, the patient’s tumor invaded the orbit and compressed the optic nerve, making it difficult for conventional radiotherapy to achieve tumor control while preserving visual function. Through meticulous planning of seed placement and quantity, we delivered a lethal radiation dose to the tumor while maintaining the optic nerve dose within its tolerance limit (18). Follow-up at one year showed no evidence of visual impairment or radiation injury to brain tissue, confirming the safety advantage of this technique in critical anatomic locations. To our knowledge, it is the first report of I-125 brachytherapy to offer a safe, effective, and repeatable therapeutic option for patients with recurrent LGMFS of the maxillary sinus who are unable to tolerate repeat surgery or chemoradiotherapy.

This case report demonstrates the potential feasibility of I-125 seed implantation for recurrent maxillary sinus LGMFS with orbital involvement, showing encouraging initial results at 12-month follow-up. However, several important limitations must be emphasized: (1) the short follow-up period precludes conclusions about long-term efficacy and late toxicity; (2) as a single case, generalizability remains uncertain; and (3) optimal patient selection criteria are yet to be established. While our preliminary results suggest potential benefits in anatomically challenging cases where conventional approaches are limited, larger prospective studies with extended follow-up periods are essential to establish the role of I-125 brachytherapy in managing recurrent LGMFS.

## 4 Conclusion

Iodine-125 seed implantation represents a promising treatment option for recurrent maxillary sinus LGMFS, particularly in cases where anatomical constraints or limitations of conventional approaches exist. Multicenter studies with longer follow-up periods are needed to establish optimal patient selection criteria and confirm long-term efficacy.

## Data Availability

The original contributions presented in the study are included in the article/**Supplementary Material**. Further inquiries can be directed to the corresponding author.
