# Supplementary material for: Iodine-125 brachytherapy for orbital-invasive low-grade myxofibrosarcoma of the maxillary sinus: a case report challenging conventional therapeutic paradigms
Source: Front Oncol. 2025 Oct 15;15:1590061. doi: 10.3389/fonc.2025.1590061 (PMC12568330; doi:10.3389/fonc.2025.1590061)
Supplement: Supplementary file 1 [file Supplementaryfile1.docx]

Supplementary Material


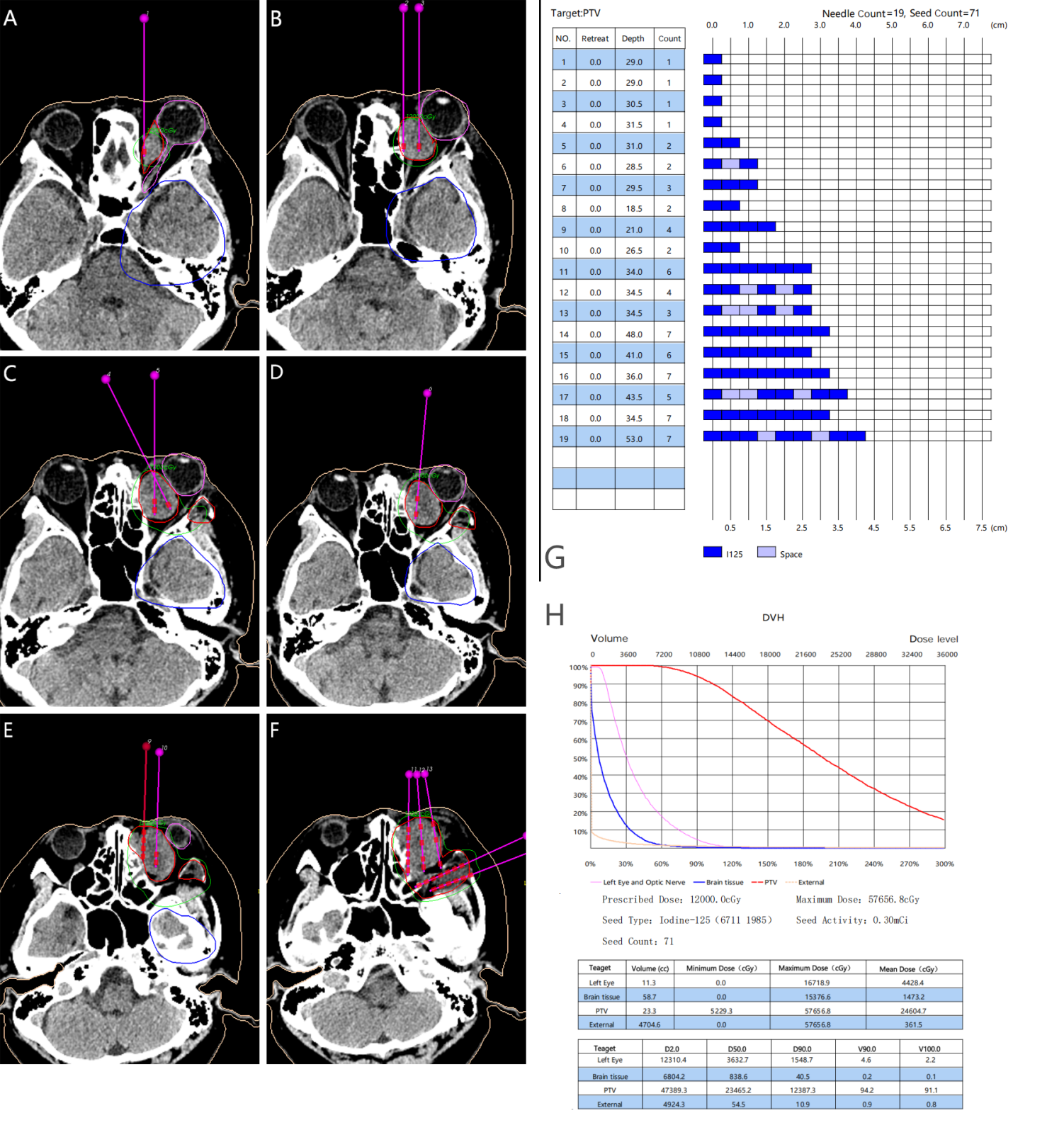


**Supplementary Figure 1.** The patient was diagnosed with low-grade myxofibrosarcoma of the maxillary sinus. For the first time preoperative treatment plan, the Treatment Planning System (TPS) prescribed a total dose of 12,000 cGy. **（A-F）**Target volume contouring and needle trajectory planning on preoperative CT images.(G) Preoperative TPS plan for seeds distribution. (H)Preoperative dosimetric evaluation (D90=12387.3cGy).


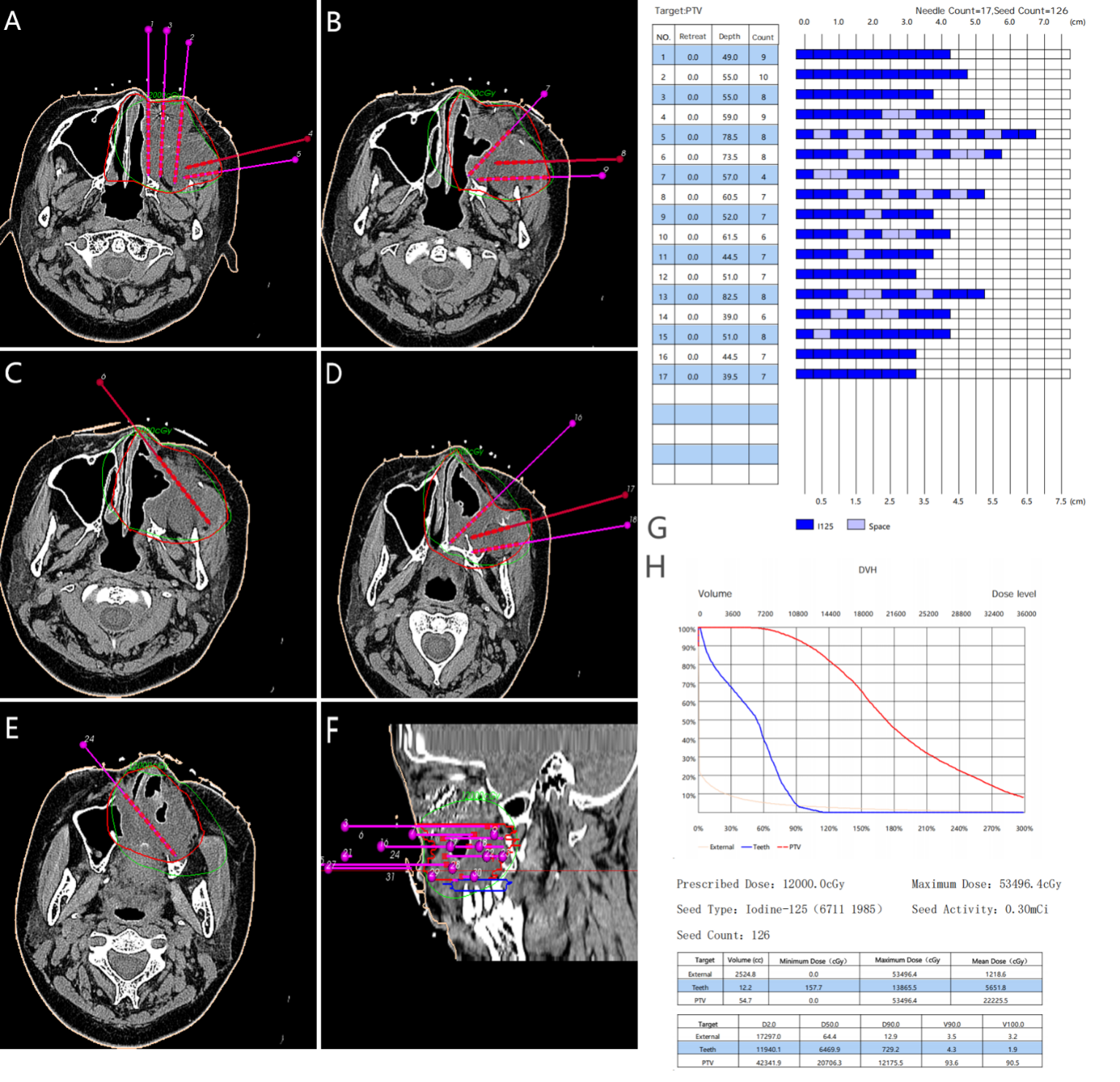


**Supplementary Figure 2.** The patient was diagnosed with low-grade myxofibrosarcoma of the maxillary sinus. For the Second time preoperative treatment plan, the Treatment Planning System (TPS) prescribed a total dose of 12,000 cGy. **（A-F）**Target volume contouring and needle trajectory planning on preoperative CT images.(G) Preoperative TPS plan for seeds distribution. (H)Preoperative dosimetric evaluation (D90=12175.5cGy).


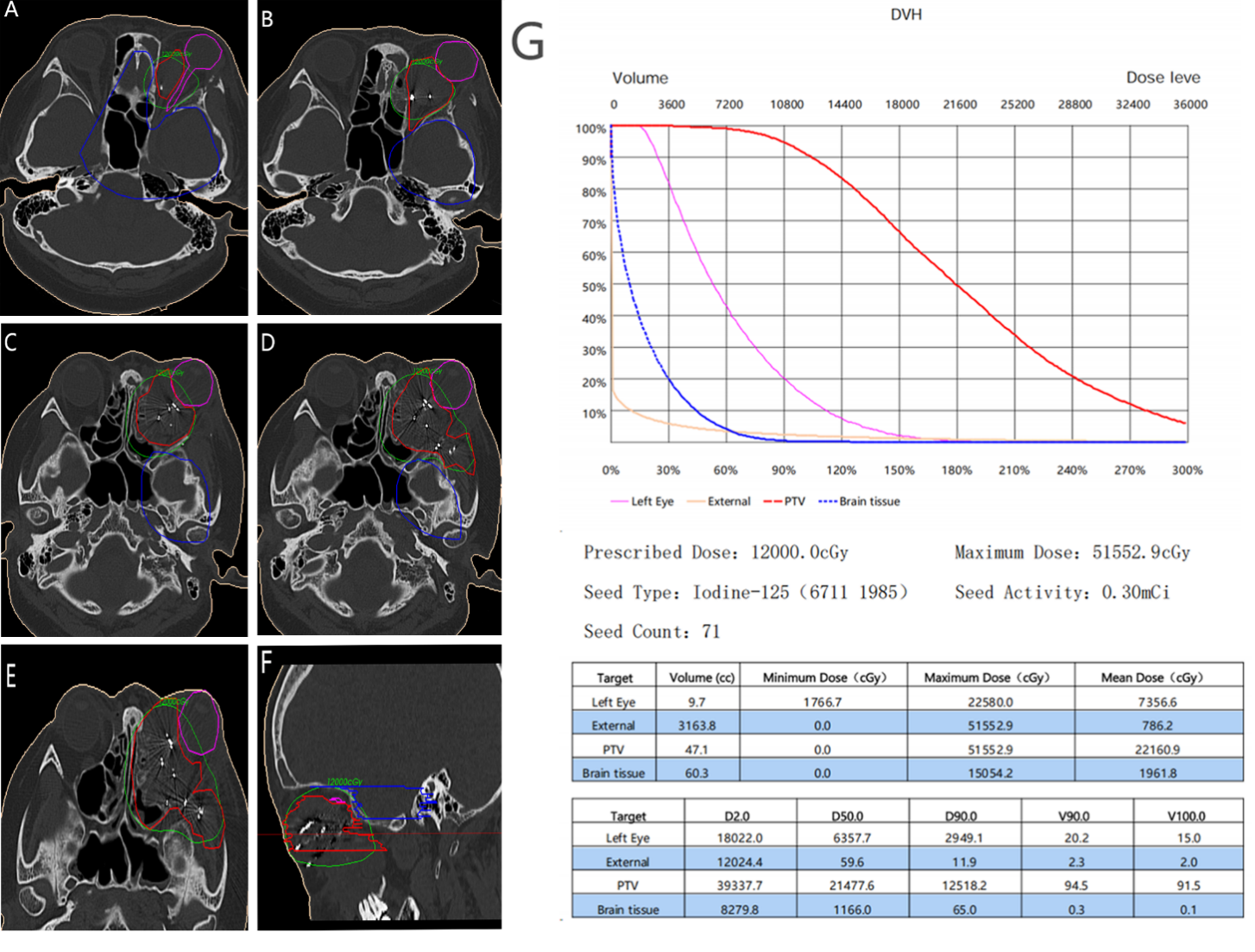


**supplemental Figure 3** Fist time postoperative CT imaging with subsequent dosimetric evaluation: (A–F) CT scan images, (G) Postoperative dosimetric evaluation (D90=12518.2cGy).


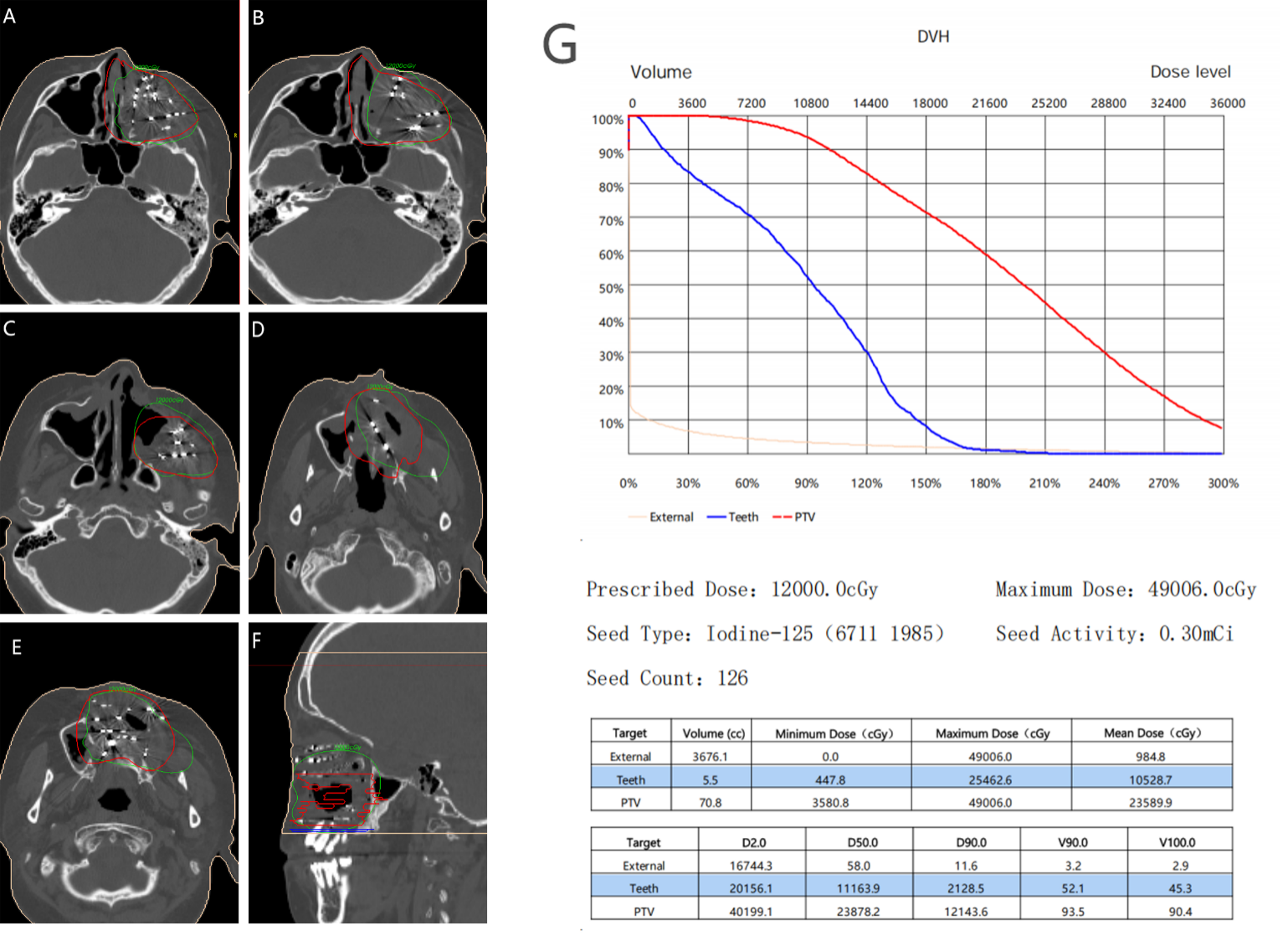


**supplemental Figure 4** Second time postoperative CT scan followed by dosimetric evaluation: (A–F) CT scan images, (G) Postoperative dosimetric evaluation (D90=12143.6cGy).
